# Piezo-phototronic effect enhanced UV photodetector based on CuI/ZnO double-shell grown on flexible copper microwire

**DOI:** 10.1186/s11671-016-1499-1

**Published:** 2016-06-03

**Authors:** Jingyu Liu, Yang Zhang, Caihong Liu, Mingzeng Peng, Aifang Yu, Jinzong Kou, Wei Liu, Junyi Zhai, Juan Liu

**Affiliations:** Beijing Institute of Nanoenergy and Nanosystems, Chinese Academy of Sciences; National Center for Nanoscience and Technology (NCNST), Beijing, 100083 People’s Republic of China; College of Environmental Sciences and Engineering, Peking University, Beijing, China

**Keywords:** Photodetector, Flexible nanodevice, Heterojunction, Piezo-phototronic effect, Double-shell nanostructure

## Abstract

In this work, we present a facile, low-cost, and effective approach to fabricate the UV photodetector with a CuI/ZnO double-shell nanostructure which was grown on common copper microwire. The enhanced performances of Cu/CuI/ZnO core/double-shell microwire photodetector resulted from the formation of heterojunction. Benefiting from the piezo-phototronic effect, the presentation of piezocharges can lower the barrier height and facilitate the charge transport across heterojunction. The photosensing abilities of the Cu/CuI/ZnO core/double-shell microwire detector are investigated under different UV light densities and strain conditions. We demonstrate the *I*-*V* characteristic of the as-prepared core/double-shell device; it is quite sensitive to applied strain, which indicates that the piezo-phototronic effect plays an essential role in facilitating charge carrier transport across the CuI/ZnO heterojunction, then the performance of the device is further boosted under external strain.

## Background

Wurtzite-structured zinc oxide and gallium nitride are gaining much attention due to their exceptional optical, electrical, and piezoelectric properties which present potential applications in the diverse areas including field effect transistors, energy harvesters, photodetectors, solar cells, pressure sensors, and chemical sensors [1–14]. In particular, one-dimensional (1D) ZnO micro/nanowires have attracted great attention due to its advantages in facile synthetic procedure, long-term chemical stability, and environmental friendliness. Coupling the semiconducting and piezoelectric properties of ZnO, applying force/strain along the ZnO polar c-axis can generate piezoelectric polarization-induced piezopotential at two ends of 1D micro/nanowire, which facilitates the modulation of charge carrier transportation process [2]. By endowing nanostructured ZnO devices with flexible capability, force/strain-modulated photoresponsing behaviors resulted from the change of Schottky barrier height (SBH) at the metal-semiconductor heterojunction or the modification of the band diagram of semiconductor composites [15, 16]. Piezo-phototronic effect utilizes external stress-induced piezopotential to modulate photo-generated carrier transport, which has promising applications for pressure-triggered sensors and human-machine interfacing [17]. The presence of piezocharges at one side of the heterojunction can considerably regulate the behaviors of the charge carriers and tune performances of flexible electronic and optoelectronic devices by controlling external strain conditions.

Recently, considerable attention has been paid to piezotronic and piezo-phototronic nanodevices based on ZnO micro/nanowires array and their strain-modulated photoresponsing behaviors [18, 19]. However, it should be noted that the presentation of free electrons in ZnO can partially screen piezopocharges under applied strain which unavoidably reduce the performances of piezotronic and piezo-phototronic nanodevice [20, 21]. In order to suppress electron screening effect, substantial efforts had already been devoted to the synthesis of p-type ZnO or the formation of heterojunction. Previous work reported the improved performance of ZnO/Au Schottky junction-based UV detector [10] because the Fermi energy of Au is lower than that of ZnO, which facilitated electron transfer from the ZnO to Au via the Schottky junction. Under external strain, the enhanced photocurrent is related to charge redistribution which induced by piezoelectric polarization at the ZnO/Au heterojunction. Another strategy for efficient separation of photo-generated electrons to prevent charge carrier recombination is the formation of semiconductor heterojunction. Involving multi-step synthetic processes for constructing complex nanostructures, several reports exhibited enhanced photoresponse of ZnO nanowires achieved with CdS or Cu_2_O under strain conditions [19, 22]. It can avoid the problems in the synthesis of p-type ZnO, which are known as the low solubility of dopants or poor stability [23]. Nevertheless, the as-fabricated photodetectors (PDs) exhibited the photoresponse in the visible region as well, which considerably influenced the direct detection of UV light. Therefore, p-type semiconductor with band gap close to ZnO can be considered as the idea building block for the formation of heterojunction and UV detector.

The Cu microwire-based devices have been reported such as piezoelectric nanogenerator, dye-sensitized solar cells, and directional gas collection/transportation [24–26]. The surface modification of Cu microwire with unique morphologies or nanostructured functional materials extensively broadened practical application in flexible devices. In this work, we present a facile and environmentally friendly approach for fabricating a flexible UV photodetector with a Cu/CuI/ZnO core/double-shell structure. The performances of Cu/CuI/ZnO microwire PD can be tuned by changing strain and illumination conditions, which indicated this type of PD has a potential application in flexible nanodevices. The CuI/ZnO heterojunction plays an essential role in modulating the photo-generated charge carrier transport with regard to the band alignment of wide band gap CuI and ZnO. Compared to the strain-free conditions, applying compressive strains leads to the photoresponsivity, relative changes of photoresponsivity, and the detectivity of Cu/CuI/ZnO photodetector was improved at different light intensities. Our work might provide an alternative strategy for utilizing piezo-phototronic effect for boosting photoresponsing performance of PD in a low-cost and convenient manner.

## Methods

The preparation of inner shell CuI was conducted by exposing the Cu microwire surface to iodine vapor. The outer shell ZnO was grown by a standard RF magnetron sputter deposition using a high purity ZnO target (99.99 %) and Ar/O_2_ (flow rate was fixed at 8:1) as the sputtering gases at room temperature. The morphology of the Cu microwire, Cu/CuI core/shell microwire, and Cu/CuI/ZnO core/double-shell microwire was examined by scanning electron microscope (SEM, Hitachi 8020). The X-ray diffraction (XRD) spectra of Cu/CuI and Cu/CuI/ZnO microwire were recorded on a Bede D1 ZM-SJ-001 diffractometer. The X-ray photoelectron spectroscopy (XPS) characterization was performed at Thermo Scientific ESCALAB 250Xi. Ag paste was employed to fix the end of Cu/CuI/ZnO core/double-shell microwire on the polyethylene terephthalate (PET) flexible substrate as electrodes. A thin layer of polydimethylsiloxane (PDMS) was used to protect the device during the test. The measurements of photoresponse properties were conducted by a semiconductor characterization system (Keithley 4200 SCS).

## Results and Discussion

**Fig. 1:**
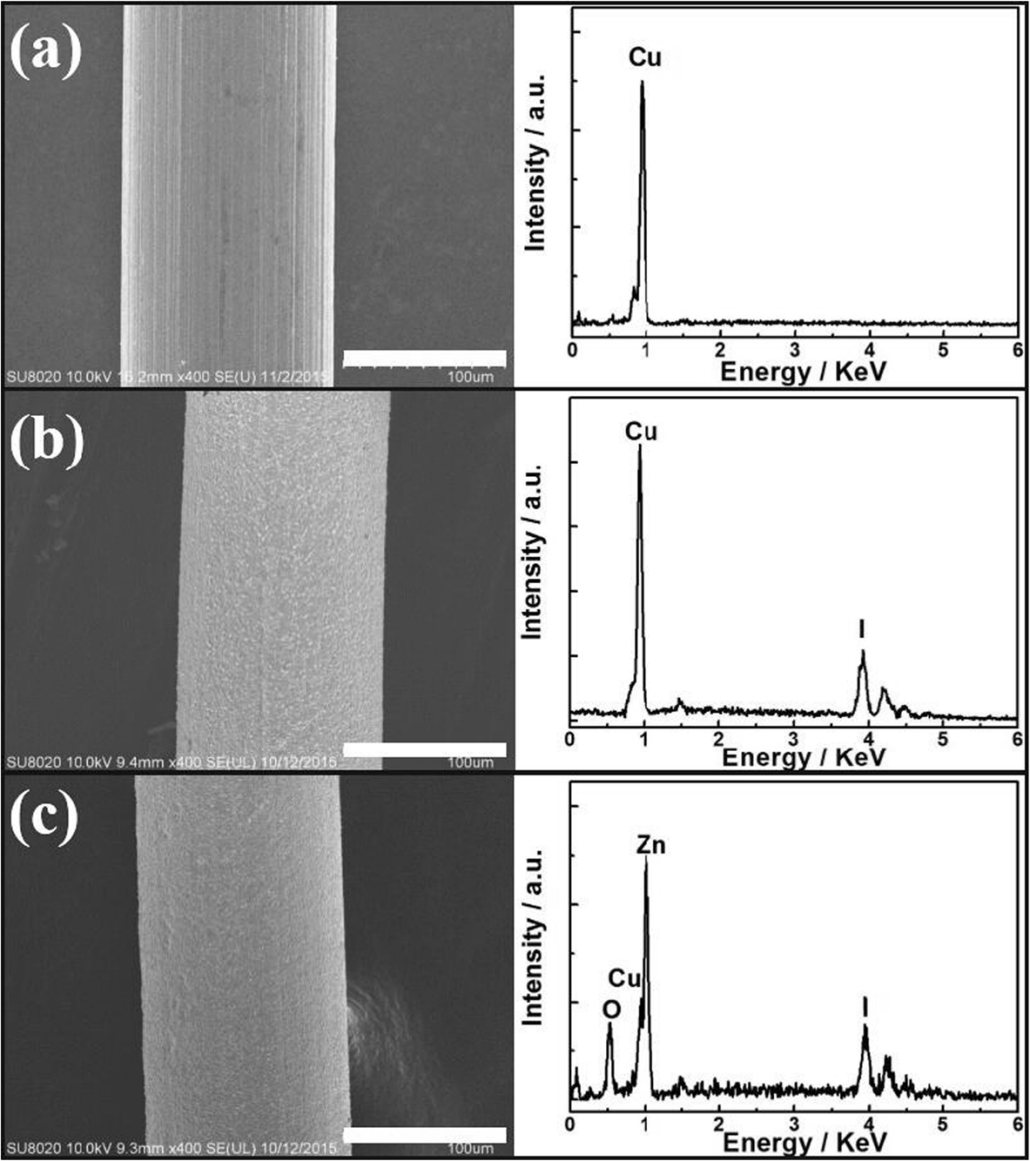
SEM images and EDS characterizations of **a** Cu microwire, **b** Cu/CuI core/shell microwire, and **c** Cu/CuI/ZnO core/double-shell microwire; all *scale bars* are 100 μm

**Fig. 2:**
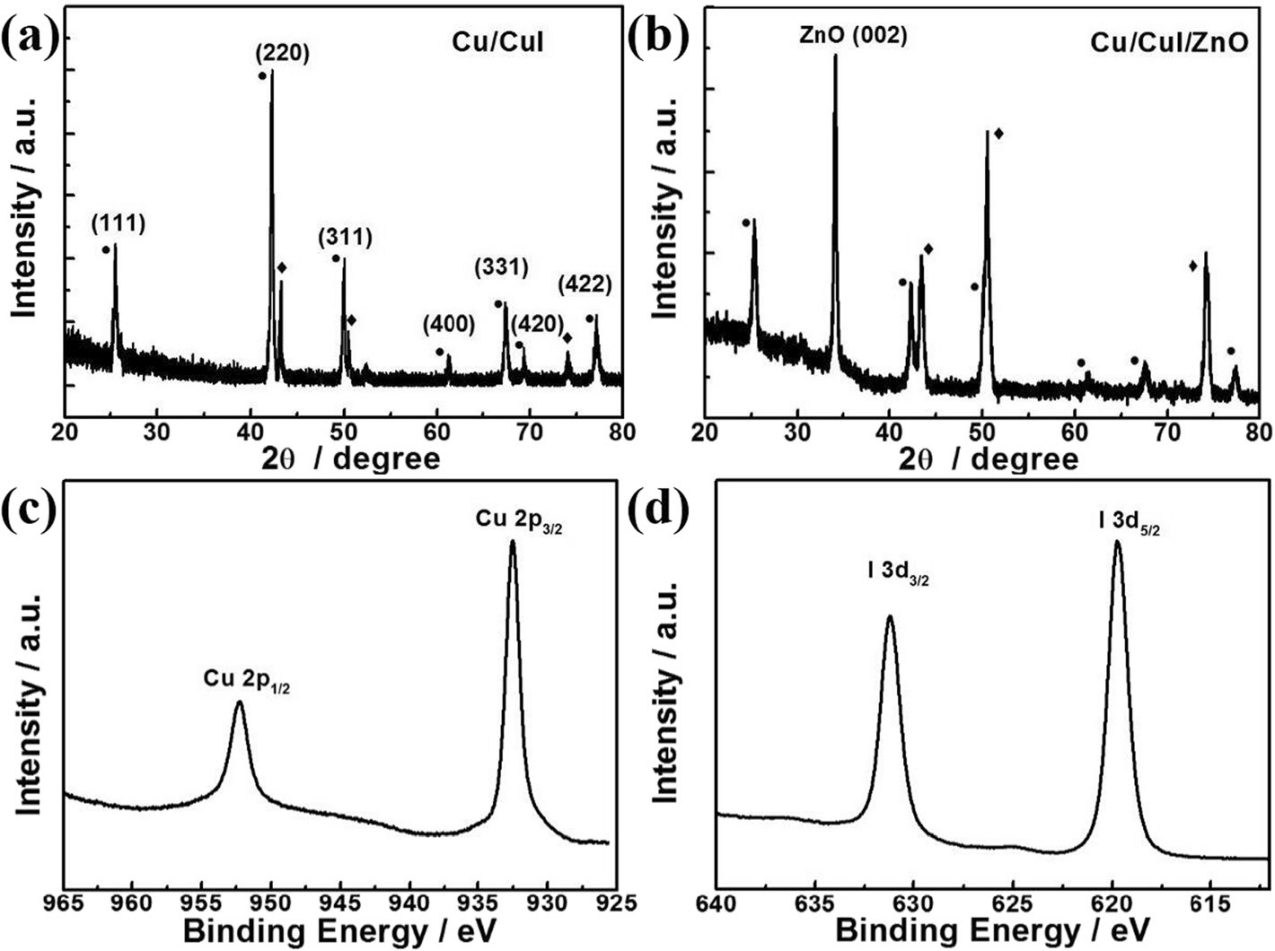
XRD spectra of **a** Cu/CuI core/shell microwires and **b** Cu/CuI/ZnO core/double-shell microwires. *Filled circle* represents CuI signals and *filled diamond* represents Cu signals. **c**, **d** XPS spectra of Cu/CuI core/shell microwire

The SEM images and EDX spectra of Cu microwire, CuI/ZnO core/shell microwire, and Cu/CuI/ZnO core/double-shell microwire are presented in Fig. 1. After carrying out one-step iodization procedure, a uniform gray-white CuI shell was observed on surface of Cu microwire, see Fig. 1b. As shown in Fig. 1c, outer shell ZnO was deposited on the top of CuI layer by a standard RF magnetron sputter deposition subsequently. For a bare Cu microwire, only Cu signals were detected in the energy dispersive spectroscopy (EDS), see Fig. 1a. As shown in Fig. 1b, iodine signals were unambiguously identified for Cu/CuI core/shell microwire. Furthermore, the presentation of ZnO coating on the top surface of Cu/CuI microwire was further confirmed by the EDS characterization, as seen in Fig. 1c. The crystal structure of Cu/CuI core/shell microwire and Cu/CuI/ZnO core/double-shell microwire was studied by XRD measurement; see Fig. 2. It can be seen from Fig. 2a that three peaks can be assigned to (111), (200), and (220) planes of face-centered cubic (fcc) Cu, which were labeled by filled circle. After ionization process, the surface of Cu microwire was fully covered by CuI shell. The XRD spectrum of Cu/CuI core/shell microwires reveals characteristic (111), (220), (311), (400), (331), (420), and (422) peaks, which indicating a cubic phase γ-CuI structure, which were labeled by filled diamond in Fig. 2b [27]. It should be mentioned that no signal belonging to Cu_2_O or CuO was detected in the XRD spectra. In order to determine the composition and valence states of the as-prepared inner shell CuI, we performed XPS study. As shown in Fig. 2c, d, the peak positions of Cu 2p_1/2_, Cu 2p_3/2_, I 3d_3/2_, and I 3d_5/2_ peaks were consistent with previous reports. According to the literature, the well-fitted single peak of I 3d_3/2_ and 3d_5/2_ signals indicates I^−^ as the only type of iodine presented in the as-prepared CuI [28, 29]. More importantly, no Cu^2+^ signal was found in the XPS spectrum, which agree with our XRD characterization. The EDS, XRD, and XPS characterization demonstrates the Cu sample was not oxidized during the sample preparation.

**Fig. 3:**
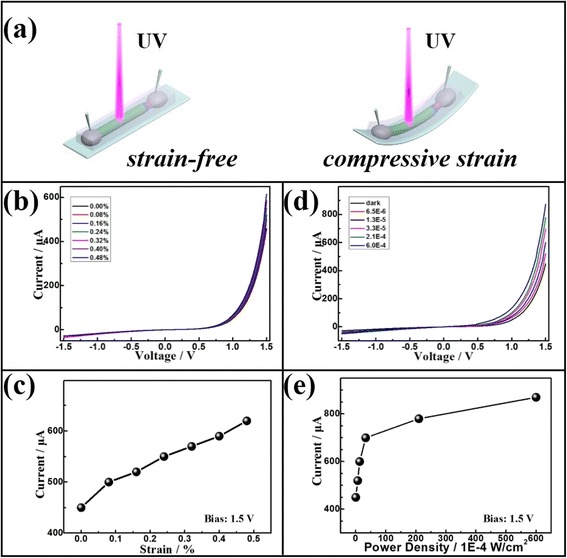
**a** Schematic illustration of the Cu/CuI/ZnO core/double-shell microwire PD; **b** *I*-*V* curves of Cu/CuI/ZnO PD under different compressive strains without illumination; **c** the enhanced output currents with the external strains, biased at 1.5 V; **d** *I*-*V* curves of Cu/CuI/ZnO core/double-shell microwire PD under different illumination power without external strain; **e** the photocurrents change with different light power, biased at 1.5 V

A description of the fabrication procedure of an individual Cu/CuI/ZnO core/double-shell microwire PD was presented in experimental section. The schematic illustration of as-prepared PD was exhibited in Fig. 3a. The Cu/CuI/ZnO core/double-shell microwire PD was fixed on the PET substrate. Compressive strain was applied to the Cu/CuI/ZnO microwire PD by bending flexible PET substrate with a three-dimensional (3D) mechanical stage and calculated according to the previous studies [19, 30]. In order to investigate the *I*-*V* characteristics of Cu/CuI/ZnO microwire PD, the device was repeatedly tested under different strain and illumination conditions, respectively. Figure 3b demonstrates that the output currents of the Cu/CuI/ZnO microwire PD can be tuned by different external strains under dark condition. In Fig. 3c, the enhanced output signals of Cu/CuI/ZnO PD has reached its maximum value at compressive strain up to 0.48 % as a 1.5 V bias is applied. It can be understood that the external stress to the PET substrate resulted in the compressive strain state of the Cu/CuI/ZnO core/double-shell microwire. The positive piezocharges generated at the region of CuI/ZnO heterojunction which modulated the charge carrier transport and will be discussed in more details below. As shown in Fig. 3d, *I*-*V* curves of Cu/CuI/ZnO microwire PD under UV light (365 nm) illumination is presented in strain-free condition, which reveals the enhanced photocurrent of Cu/CuI/ZnO microwire PD was observed by increasing the power densities of the illumination; see Fig. 3e. It can be concluded that increasing compressive strain and illumination power can effective influence the response behaviors of Cu/CuI/ZnO core/double-shell microwire device, respectively.

**Fig. 4:**
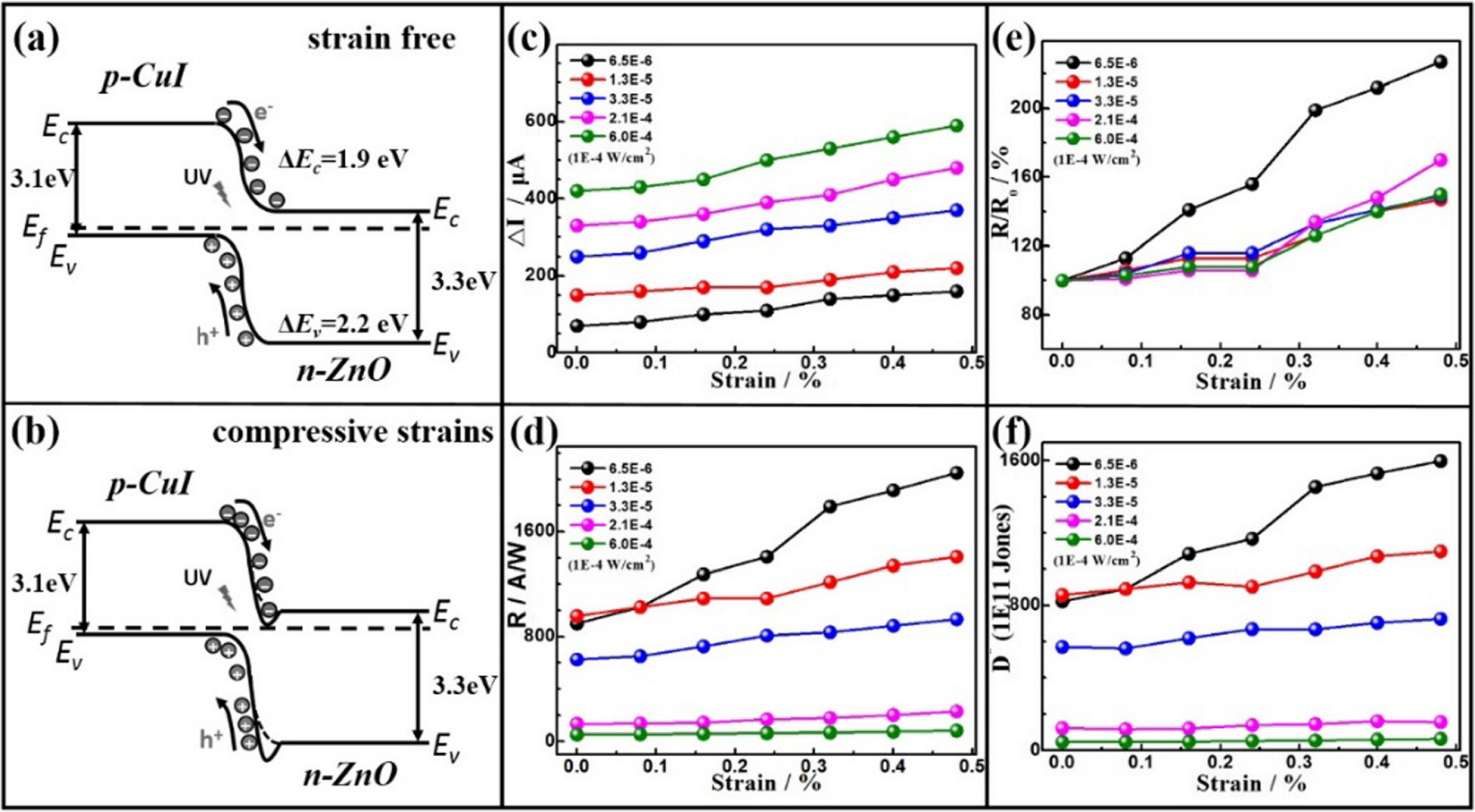
Schematic band diagrams of a CuI/ZnO heterojunction **a** without and **b** with compressive strain to illustrate the working mechanism of piezo-phototronic effect-enhanced PD performance; **c** photocurrent ΔI, **d** photoresponsivity *R*, and **e** relative changes of photoresponsivity *R/R*_0_ and **f** the detectivity D* of CuI/ZnO PD under different strain and illumination conditions, biased at 1.5 V

According to the previous reports, the relative band positions in CuI/ZnO may play a substantial role in achieving high responsivity of photodetector. The impact of compressive strain on the change of output currents in the Cu/CuI/ZnO microwire PD can be explained through their band diagrams; see Fig. 4a. The electron affinity of CuI and ZnO are χ(CuI) = 2.1 eV and χ(ZnO) = 4.0 eV, and the band gap of CuI and ZnO are *E*_g_(CuI) = 3.1 eV and *E*_g_(ZnO) = 3.3 eV, respectively. After the formation of CuI/ZnO heterojunction, a conduction band (CB) offset Δ*E*_c_ = χ(ZnO) − χ(CuI) = 1.9 eV and a valence band (VB) offset Δ*E*_v_ = *E*_g_(ZnO) − *E*_g_(CuI) + Δ*E*_c_ = 2.1 eV is presented at the heterojunction [31]. Externally triggered by UV light, the photoexcited electrons on the CB of inner shell CuI can be transferred to outer shell ZnO because the CB and VB of ZnO lie below the energy band of CuI, while the excited holes on the VB of outer ZnO layer simultaneously transfer to inner CuI layer. Driven by the build-in field at the CuI/ZnO heterojunction, the photo-generated electrons and holes can be largely separated, which further increases the photocurrent. However, taking the compressive strain into consideration, the positive piezocharges are generated at ZnO side of heterojunction, which lower the barrier height and result in the depletion region slightly shifting toward the side of CuI. It can be expected that the separation of photo-generated electron-hole pairs at the CuI/ZnO heterojunction is further promoted by simultaneously increasing UV illumination power and compressive strain. Based on our experimental data, the merit of piezo-phototronic effect which enhanced performance of Cu/CuI/ZnO microwire PD was considerably highlighted. However, the positive piezocharges which mechanically generated by the ZnO still have possibility to trap a small amount of the photo-generated electrons; see Fig. 4a, b. It should be declared that the output current may slightly decrease due to CuI/ZnO interface defects.

To evaluate the photosensing performance of the flexible Cu/CuI/ZnO core/double-shell microwire PDs, the as-fabricated device was measured by increasing compressive strain and the power of UV light. The change of photocurrent ΔI (ΔI = I-I_dark,s_, I_dark,s_ represents the corresponding dark current under certain strains) are summarized in Fig. 4c. Under each illumination conditions, the photocurrents increase with raising compressive strains from 0 to 0.48 %. The strain-modulated photoresponse behaviors of Cu/CuI/ZnO core/double-shell microwire PD can be understood by piezo-phototronic effect. The detailed discussion will require further analysis of the key factors of photoresponsivity *R* and relative changes of photoresponsivity *R* (compressive strain conditions) with respect to *R*_0_ (strain-free conditions) and detectivity D*, which are calculated according to the previous method; see Fig. 4d−f [19]. The *R*, *R*/*R*_0_ and detectivity (D*) values were recorded at different illumination and strain conditions with the electrode 1.5 V biased. We found that the improved performance of the Cu/CuI/ZnO microwire PDs can be achieved by gradually increasing compressive stain. The maximum values of *R*, *R*/*R*_0_, and D* are 2050, 227 %, and 1.597 × 10^14^ Jones which obtained under a compressive strain (0.48 %) with an illumination condition (6.5 × 10^−6^ W/cm^2^). It can be understood that the mechanically generated piezocharges at the region of heterojunction plays a central role in enhancing the performance of PD under UV illumination. For low intensity UV illumination, the photoexcited electron-hole pairs are almost completely separated and redistributed by the build-in electric field, which the photocurrents were largely boosted. At the CuI/ZnO heterojunction, the existence of piezocharges can reduce the barrier height and then increase the photocurrent. While the high intensity UV illumination on the CuI/ZnO heterojunction resulted a large number of electron-hole pairs are photoexcited, the recombination of electron-hole results in photocurrent trends to saturation. Consequently, the small amount of piezocharges at heterojunction cannot further increase the photocurrent. The strain-induced piezocharges played an important role in enhancing the performance of piezotronic and piezo-phototronic effect based nanodevice [5]. However, the free electrons in outer shell ZnO can partly screen piezocharges. The employment of p-type CuI as inner shell can effectively reduce the amount of free electrons in the ZnO. Therefore, more piezocharges can be effectively utilized by applying compressive strain to the Cu/CuI/ZnO core/double-shell microwire PD. Basing on the principle of piezo-phototronics, it can be concluded that piezocharges play an imperative role in modulating CuI/ZnO photoresponsing performance at a low light intensity and large strain conditions.

## Conclusions

We have developed a convenient approach for preparing piezo-phototronic effect enhanced UV photodetector with Cu/CuI/ZnO core/double-shell microwire structure. The photoresponsing behaviors of the Cu/CuI/ZnO microwire can be controllably tuned by strain and illumination conditions, respectively. In comparison to other narrow band gap semiconductors used to build UV photodetector, wide band gap make CuI a promising candidate for avoiding unfavorable influence from visible light. Furthermore, inner shell CuI plays a significant role in modulating the transport of charge carrier across the heterojunction under UV illumination due to the staggered band alignment of CuI and ZnO. Under strain conditions, piezocharges are mechanically generated at the ZnO side of heterojunction which has an essential impact on the depletion width. Compared to the strain-free condition, applying compressive strains lead to the photoresponsivity, relative changes of photoresponsivity, and the detectivity of Cu/CuI/ZnO microwire photodetector was largely improved at different illumination conditions. This work provides a reliable and inexpensive method for utilizing piezo-phototronic effect for boosting the performance of UV photodetector with a low-cost, environmentally friendly and convenient technique.

## Abbreviations

1D, one-dimensional; 3D, three-dimensional; CB, conduction band; EDS, energy dispersive spectroscopy; PDMS, polydimethylsiloxane; PDs, photodetectors; PET, polyethylene terephthalate; SBH, Schottky barrier height; SEM, scanning electron microscope; UV, ultraviolet; VB, valence band; XPS, X-ray photoelectron spectroscopy; XRD, X-ray diffraction

## References

[1] Wang ZL, Song J (2006). Piezoelectric nanogenerators based on zinc oxide nanowire arrays. Science.

[2] Wang ZL (2012). Progress in piezotronics and piezo-phototronics. Adv Mater.

[3] Shi J, Zhao P, Wang X (2013). Piezoelectric-polarization-enhanced photovoltaic performance in depleted-heterojunction quantum-dot solar cells. Adv Mater.

[4] Xu Z, Zhang C, Wang WL, Bando Y, Bai XD, Golberg D (2015). Lateral piezopotential-gated field-effect transistor of ZnO nanowires. Nano Energy.

[5] Sohn JI, Cha SN, Song BG, Lee S, Kim SM, Ku J, Kim HJ, Park YJ, Choi BL, Wang ZL, Kim JM, Kim K (2013). Engineering of efficiency limiting free carriers and an interfacial energy barrier for an enhancing piezoelectric generation. Energy Environ Sci.

[6] Liu W, Zhang AH, Zhang Y, Wang ZL (2015). First principle simulations of piezotronic transistors. Nano Energy.

[7] Zhang Y, Liu C, Liu J, Xiong J, Liu J, Zhang K, Liu Y, Peng M, Yu A, Zhang A, Zhang Y, Wang Z, Zhai J, Wang ZL (2016). Lattice strain induced remarkable enhancement in piezoelectric performance of ZnO-based flexible nanogenerators. ACS Appl Mater Interfaces.

[8] Liu C, Yu A, Peng M, Song M, Liu W, Zhang Y, Zhai J (2016). Improvement in the piezoelectric performance of a ZnO nanogenerator by a combination of chemical doping and interfacial modification. J Phys Chem C.

[9] Song M, Zhang Y, Peng MZ, Zhai JY (2014). Low frequency wideband nano generators for energy harvesting from natural environment. Nano Energy.

[10] Lu S, Qi J, Liu S, Zhang Z, Wang Z, Lin P, Liao Q, Liang Q, Zhang Y (2014). Piezotronic interface engineering on ZnO/Au-based Schottky junction for enhanced photoresponse of a flexible self-powered UV detector. ACS Appl Mater Interfaces.

[11] Wang PL, Fu YM, Yu BW, Zhao YY, Xing LL, Xue XY (2015). Realizing room-temperature self-powered ethanol sensing of ZnO nanowire arrays by combining their piezoelectric, photoelectric and gas sensing characteristics. J Mater Chem A.

[12] Nie Y, Deng P, Zhao Y, Wang P, Xing L, Zhang Y, Xue X (2014). The conversion of PN-junction influencing the piezoelectric output of a CuO/ZnO nanoarray nanogenerator and its application as a room-temperature self-powered active H_2_S sensor. Nanotechnology.

[13] Du CH, Jiang CY, Zuo P, Huang X, Pu X, Zhao ZF, Zhou YL, Li LX, Chen H, Hu WG, Wang ZL (2015). Piezo-phototronic effect controlled dual-channel visible light communication (PVLC) using InGaN/GaN multiquantum well nanopillars. Small.

[14] Peng M, Liu Y, Yu A, Zhang Y, Liu C, Liu J, Wu W, Zhang K, Shi X, Kou J, Zhai J, Wang ZL (2016). Flexible self-powered GaN ultraviolet photoswitch with piezo-phototronic effect enhanced On/Off ratio. ACS Nano.

[15] Rai SC, Wang K, Ding Y, Marmon JK, Bhatt M, Zhang Y, Zhou W, Wang ZL (2015). Piezo-phototronic effect enhanced UV/visible photodetector based on fully wide band gap type-II ZnO/ZnS core/shell nanowire array. ACS Nano.

[16] Wen XN, Wu WZ, Ding Y, Wang ZL (2013). Piezotronic effect in flexible thin-film based devices. Adv Mater.

[17] Peng M, Li Z, Liu C, Zheng Q, Shi X, Song M, Zhang Y, Du S, Zhai J, Wang ZL (2015). High-resolution dynamic pressure sensor array based on piezo-phototronic effect tuned photoluminescence imaging. ACS Nano.

[18] Zhang F, Niu S, Guo W, Zhu G, Liu Y, Zhang X, Wang ZL (2013). Piezo-phototronic effect enhanced visible/UV photodetector of a carbon-fiber/ZnO-CdS double-shell microwire. ACS Nano.

[19] Wang Z, Yu R, Pan C, Liu Y, Ding Y, Wang ZL (2015). Piezo-phototronic UV/visible photosensing with optical-fiber-nanowire hybridized structures. Adv Mater.

[20] Wang ZN, Yu RM, Wen XN, Liu Y, Pan CF, Wu WZ, Wang ZL (2014). Optimizing performance of silicon-based p-n junction photodetectors by the piezo-phototronic effect. ACS Nano.

[21] Pradel KC, Wu W, Zhou Y, Wen X, Ding Y, Wang ZL (2013). Piezotronic effect in solution-grown p-type ZnO nanowires and films. Nano Lett.

[22] Lin P, Chen X, Yan XQ, Zhang Z, Yuan HG, Li PF, Zhao YG, Zhang Y (2014). Enhanced photoresponse of Cu_2_O/ZnO heterojunction with piezo-modulated interface engineering. Nano Res.

[23] Wang F, Seo JH, Bayerl D, Shi J, Mi H, Ma Z, Zhao D, Shuai Y, Zhou W, Wang X (2011). An aqueous solution-based doping strategy for large-scale synthesis of Sb-doped ZnO nanowires. Nanotechnology.

[24] Heng LP, Wang XY, Yang NL, Zhai J, Wan MX, Jiang L (2010). P-N-junction-based flexible dye-sensitized solar cells. Adv Funct Mater.

[25] Ma R, Wang JM, Yang ZJ, Liu M, Zhang JJ, Jiang L (2015). Bioinspired gas bubble spontaneous and directional transportation effects in an aqueous medium. Adv Mater.

[26] Lei JX, Yin B, Qiu Y, Zhang HQ, Chang Y, Luo YM, Zhao Y, Ji JY, Hu LZ (2015). Flexible piezoelectric nanogenerator based on Cu_2_O-ZnO p-n junction for energy harvesting. RSC Adv.

[27] Hu XL, Yu JC, Gong JM, Li Q (2007). A facile surface-etching route to thin films of metal iodides. Cryst Growth Des.

[28] Sankapal BR, Ennaoui A, Guminskaya T, Dittrich T, Bohne W, Rohrich J, Strub E, Lux-Steiner MC (2005). Characterization of p-CuI prepared by the SILAR technique on Cu-tape/n-CuInS2 for solar cells. Thin Solid Films.

[29] Saha S, Das S, Sen D, Ghorai UK, Mazumder N, Gupta BK, Chattopadhyay KK (2015). Bane to boon: tailored defect induced bright red luminescence from cuprous iodide nanophosphors for on-demand rare-earth-free energy-saving lighting applications. J Mater Chem C.

[30] Xu S, Qin Y, Xu C, Wei Y, Yang R, Wang ZL (2010). Self-powered nanowire devices. Nat Nanotechnol.

[31] Schein FL, von Wenckstern H, Grundmann M (2013). Transparent p-CuI/n-ZnO heterojunction diodes. Appl Phys Lett.

